# The Effect of Microporous Polymeric Support Modification on Surface and Gas Transport Properties of Supported Ionic Liquid Membranes

**DOI:** 10.3390/membranes6010004

**Published:** 2015-12-30

**Authors:** Alsu A. Akhmetshina, Ilsiya M. Davletbaeva, Ekaterina S. Grebenschikova, Tatyana S. Sazanova, Anton N. Petukhov, Artem A. Atlaskin, Evgeny N. Razov, Ilnaz I. Zaripov, Carla F. Martins, Luísa A. Neves, Ilya V. Vorotyntsev

**Affiliations:** 1 Nizhny Novgorod State Technical University n.a. R.E. Alekseev, 24 Minina str., Nizhny Novgorod 603950, Russia; aai-89@mail.ru (A.A.A.); davletbaeva09@mail.ru (I.M.D.); yarymova.tatyana@yandex.ru (T.S.S.); fox-off@mail.ru (A.N.P.); atlaskin@gmail.com (A.A.A.); 2 Kazan National Research Technological University, 68 Karl Marks str, Kazan 420015, Russia; zaripovilnaz@gmail.com; 3 Kazan Federal University, 18 Kremlyovskaya St., Kazan 420008, Russia; jewelery_77@mail.ru; 4 Institute for Problems in Mechanical Engineering, Russian Academy of Sciences, 85 Belinskogo str., Nizhny Novgorod 603024, Russia; razov_e@mail.ru; 5 LAQV, REQUIMTE, Departamento de Química, Faculdade de Ciências e Tecnologia, Universidade Nova de Lisboa, 2829-516 Caparica, Portugal; cmm17205@campus.fct.unl.pt (C.F.M.); luisa.neves@fct.unl.pt (L.A.N.)

**Keywords:** microporous polymers, modification, ionic liquid, supported liquid membrane, SILMs, gas separation

## Abstract

Microporous polymers based on anionic macroinitiator and toluene 2,4-diisocyanate were used as a support for 1-butyl-3-methylimidazolium hexafluorophosphate ([bmim][PF_6_]) and 1-ethyl-3-methylimidazolium bis(trifluoromethylsulfonyl)imide ([emim][Tf_2_N]) immobilization. The polymeric support was modified by using silica particles associated in oligomeric media, and the influence of the modifier used on the polymeric structure was studied. The supported ionic liquid membranes (SILMs) were tested for He, N_2_, NH_3_, H_2_S, and CO_2_ gas separation and ideal selectivities were calculated. The high values of ideal selectivity for ammonia-based systems with permanent gases were observed on polymer matrixes immobilized with [bmim][PF_6_] and [emim][Tf_2_N]. The modification of SILMs by nanosize silica particles leads to an increase of NH_3_ separation relatively to CO_2_ or H_2_S.

## 1. Introduction

Ionic liquids (ILs) are compounds composed of bulky organic cations and organic or inorganic anions which have a low melting point and are liquids at temperatures below 100 °C. In recent decades, an increase of interest in ILs has been observed due to their unique properties such as high thermal and chemical stability, non-volatility, and compatibility with various substances [1,2,3,4]. The structure of cations is based on quaternary ammonium or heterocyclic ions (imidazolium, pyridinium, pyrrolidinium, phosphonium), and the most frequently used anions are derived from organic or inorganic acidic residues such as sulfates, nitrates, acetates, sulfonates. Since their early development, several promising applications of ILs include their use as “green solvents” for various chemical processes [5,6,7], extraction solvents [8,9], electrochemical devices such as ion batteries [10,11], fuel cells [12,13,14,15], solar cells [16,17], and gas separation processes [18,19,20,21,22]. The amphiphilic nature of ILs is easily observed by the intense interactions between IL species and polar or nonpolar substances. These characteristics make the application of ILs as sorbents for gases and in membrane separation technology of extreme interest.

The removal of acidic gases such as CO_2_ and H_2_S is an essential stage of natural gas and biogas treatment. Among the traditional methods commercially available for acidic gas separation, the most common methods include absorption by physical and chemical absorbents (e.g., amine solutions, carbonates), cryogenic distillation, membrane separation, and catalytic oxidation [23]. In general, all these methods have a specific field of application limited by the sorption capacity, regeneration, and stability to high pressures. The novel trend in gas stream treatment is judicious selection of enhanced solvents for aggressive gases based on sorption of H_2_S and CO_2_ using ionic liquids.

The sorption of ammonia by ionic liquids was reported for imidazolium-based [24] and guanidinium-based [25] cations paired with different anions, including tetrafluoroborate, hexafluorophosphate, bis(trifluoromethylsulfonyl)imide, nitrate, trifluoromethanesulfonate, chloride, acetate, and thiocyanate, however this approach has also been used in some specific applications using membrane contactors [26,27]. The high solubility of ammonia in ILs shows promise for the development of ammonia treatment systems, replacing the traditional ammonia-water system.

The membranes with immobilized ILs, which are held into support by capillary forces, are traditionally referred to as supported ionic liquid membranes (SILMs). Since their development, several different methods of SILM preparation have been reported, namely penetration of IL into the membrane by direct immersion [28], vacuum [29], pressure [30] or their combination, and fabrication of composite materials [31]. Each method carries its own advantages and disadvantages which affect the thermal, mechanical and operational properties of the membrane. The complete filling of the porous support with IL is unsuitable by immersion methods due to the presence of air in the voids of the membrane; given this, preliminary removal of air from the pores of the support is necessitated [22]. The immobilization of IL by vacuum or pressure solves the aforementioned problems; however, the stability of the liquid phase on support matrix decreases with an increase in the applied pressure. In an attempt to improve the stability of SILMs, several authors proposed the fabrication of IL and polymer composites [31].

As candidates for a supported matrix, different types of microporous materials (polymeric or inorganic) can be used. Polyethersulfone [32], polyvinylidene fluoride [33,34], polytetrafluoroethylene [30], nylon [35], chitin, and chitosan [36] are employed as polymeric supports for gas separation. In previous works, the microporous polymeric material based on anionic macroinitiators and toluene 2,4-diisocyanate was synthesized [37]. The notable thermal, mechanical, and gas permeation properties for the obtained polymer was thoroughly characterized and exceptional properties were found for this material. In the current research, however, we suggest the modification of the previously studied polymer by silica clusters associated in oligomeric media (ASC). The addition of nanoparticles presents a promising route toward the formation of block-structured organic-inorganic polymers which are expected to influence the segregation of polymer blocks and the porous structure of polymeric material.

Seeing this impressive precedent work has inspired the current study toward the preparation of SILMs based on the microporous polymeric materials and 1-butyl-3-methylimidazolium hexafluorophosphate and 1-ethyl-3-methylimidazolium bis(trifluoromethylsulfonyl)imide ionic liquids for removal of hazardous gases like ammonia, hydrogen sulfide, and carbon dioxide.

## 2. Results and Discussion

A unique feature of the polymer matrix is the presence of rigid O-polyisocyanate components. In addition, an amphiphilic block copolymer (PPEG) with a relative high molecular weight is another structural element of the polymer which represents an area of intense interest. Previously, the reaction of toluene 2,4-diisocyanate with macroinitiators was studied, in which the block copolymers of propylene and ethylene oxide (PPEG) containing terminal hydroxyl groups are partially replaced by potassium alcoholate groups [37]. In numerous previous works [38,39,40], the conditions of acetal (O-polyisocyanates) and amide (N-polyisocyanates) polyisocyanates formation have been investigated to prove the result of isocyanate groups opening along the C=O or C=N bonds, respectively. These studies have elucidated that the isocyanate group opening depends strongly on the reaction conditions employed (temperature, solvent, or presence of co-catalyst).

The supramolecular structure of polymers is based on the core-shell microphase separation of flexible and rigid blocks. This process also creates the conditions for the formation of voids in the polymer matrix. The voids can be arranged regularly to have constant cross-sectional dimensions, and the diameter of cross section depends on the supramolecular arrangement of the macroinitiator (flexible amphiphilic block) and O-polyisocyanates (rigid block).

For the development of nanostructured polymers with variable pore size dimensions, silica clusters associated in oligomeric media (ASC) were prepared and investigated as modifiers of the polymeric matrix. The protocol of preparation relies on sol-gel technology, whereby the growth of silica particles is localized near by the shell of thermodynamically incompatible oligomers. As a result, clusters of silica surrounded by the chemically varied oligomers exhibit low aggregation and are soluble in organic media due to the lability of intermolecular bonds.

### 2.1. ASC Preparations and Characterization

**Figure 1:**
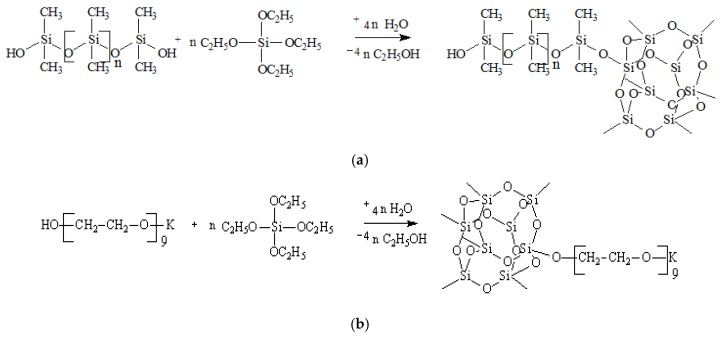
(**a**) Interaction of PDMS with TEOS involving the latent water and (**b**) interaction of PEG with TEOS involving the latent water.

**Figure 2:**
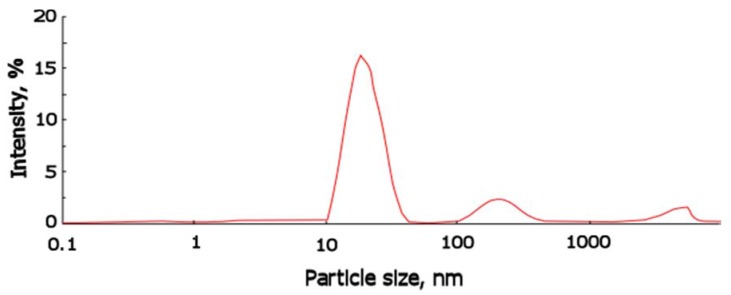
Particle size distribution for ASC obtained in presence of 0.5%_wt_ of potassium alcoholate groups and 0.05%_wt_ of water, determined by dynamic light scattering.

The raw materials used for the preparation of ASC were tetraethoxysilane (TEOS), polyethylene glycol (molecular weight 400 g/mol) (PEG), and a low molecular weight polydimethylsiloxane (PDMS). As a source of potassium alcoholate group, potassium diethylene glycol (DEG-K) was used. The modifier was synthesized by the transetherification of TEOS and subsequent polycondensation of the reaction adducts. The main steps of the synthesis are shown in Figure 1a,b:

The content of potassium was equal to 0.5%_wt_ and the water content 0.05%_wt_; ASC was isolated as a white low-viscosity liquid which is dissolved in toluene and ethyl acetate, and is stable in organic media for more than a year. The estimation of the size of silica clusters associated in oligomeric media was performed. Figure 2 shows the distribution function of the particle size for ASC in toluene determined by dynamic light scattering method.

According to the analysis of distribution function, the average size for ASC associates is equal to 20 nm. These results indicate that potassium alcoholate groups perform the significant catalytic function in the reactions of hydrolysis and subsequent polycondensation with TEOS.

### 2.2. Investigation of the Polymer Matrix Properties

**Figure 3:**
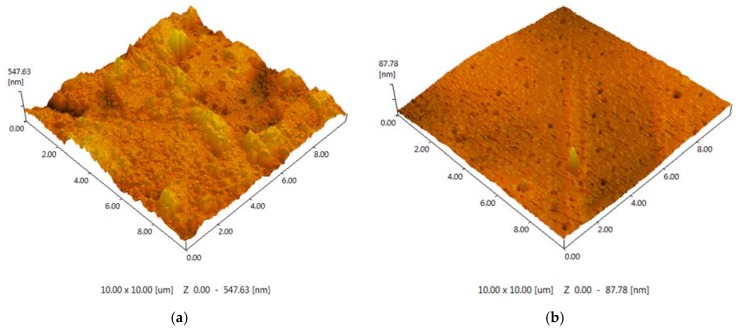
(**a**) AFM image (10 × 10 μm) for the unmodified polymer; (**b**) AFM image (10 × 10 μm) for the polymer modified by 0.8%_wt_ ASC.

The polymeric support for the ionic liquids immobilization was synthesized on the basis of toluene 2,4-diisocyanate (TDI) and propylene or ethylene oxide (PPEG). A series of samples were obtained with the use of ASC, and the molar ratio [PPEG]:[TDI] was equal to 15. In Figure 3, images of the polymeric surface obtained by atomic force microscopy are presented for the polymer based on [PPEG]: [TDI] = 1:15 and ASC modified polymer. The atomic force microscopy (AFM) images allow visualization of the pores on the surface of polymeric films and estimation of their average size. The pores were found to have the form of tapered capillaries. The diameter of the mouth of the pores was obtained from AFM images (Figure 3). The measurement of pore diameter was initiated from the first minima found [41]. 

**Figure 4:**
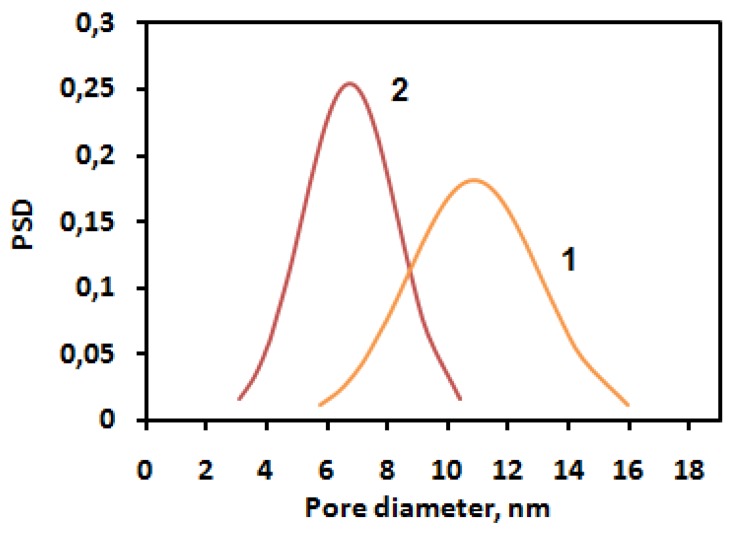
Pore size distributions for the polymer [PPEG]:[TDI] = 1:15 (co-catalyst CuCl_2_) (**1**) and ASC modified polymer (**2**).

The sample modified by ASC exhibits narrow pore size distribution with an average pore size equal to 7 nm (Figure 4). The silica nanoparticles caused a significant impact on the surface morphology. An increase of roughness for the unmodified polymer (R_a_ = 48.99 ± 0.01 nm) was found; in the case of ASC, the roughness of the polymeric surface significantly decreased (R_a_ = 3.45 ± 0.01 nm), as we can also see from the AFM image (Figure 3). According to the obtained results, the most efficient structuring of polymers occurs in the case of ASC applying with an average size of associates near the 20 nm (Figure 2).

**Figure 5:**
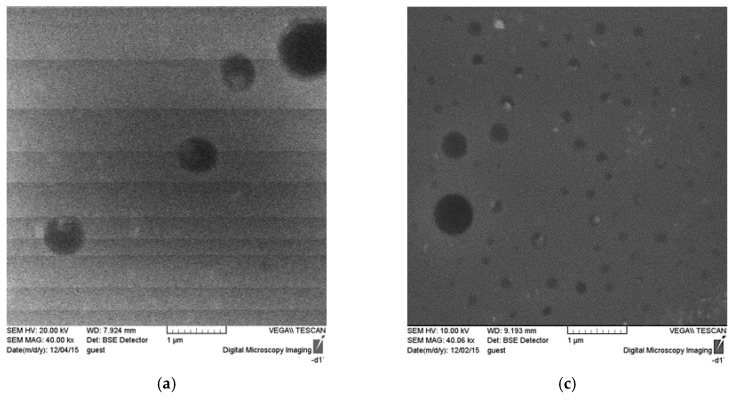

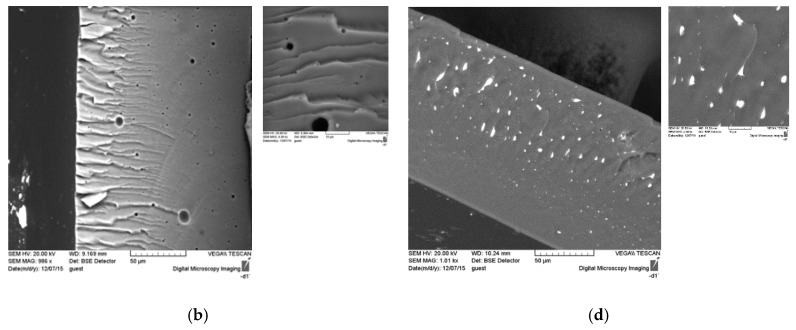
SEM images of the surface and cross section of unmodified polymer ((**a**) surface; (**b**) cross-section) and ASC modified polymer ((**c**) surface; (**d**) cross-section).

Figure 5 presents images obtained by scanning electronic microscopy (SEM). In the cross-section images, it can be observed that there is a correlation with the AFM results for the polymers. In the cross-sectional images, it is possible to observe that the membranes are practically dense with only punctual micropores.

**Figure 6:**
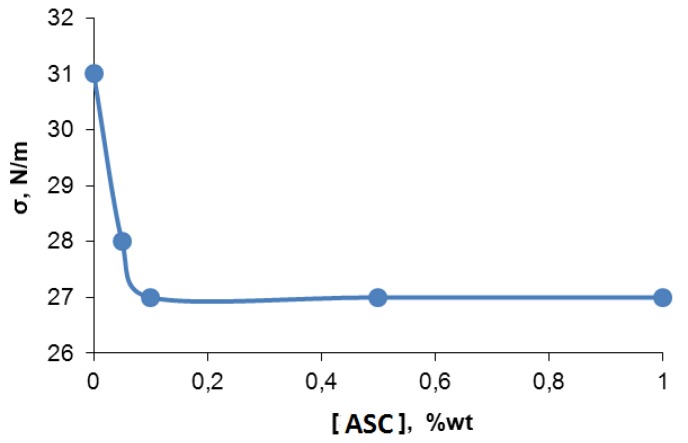
Surface tension dependence of toluene solution with ASC content.

The causes of microphase separation were enhanced under the influence of silica clusters associated in oligomeric media and may be related with its (ASC) surfactant properties. For this reason, we investigated the dependence of specific surface tension of toluene with dissolved ASC in order to validate the aforementioned hypothesis. According to Figure 6, the specific surface tension of ASC in toluene solution increases from 27 to 31 N/m with ASC concentrations decrease. 

### 2.3. Characterization of SILMs

The stability of supported ionic liquid membranes is determined by the ability to operate under pressure without weight loss. This requires that during gas separation processes, the total amount of IL immobilized should remain constant inside the pores of the support. The intensive interactions that may occur between the matrix and the IL contribute to the SILM stability, which can be further estimated using contact angle measurements between the IL and the polymeric support.

The hydrophobicity of the microporous polymers used was investigated by contact angle measurements [42]. The polymers on the basis of [PPEG]:[TDI] = 1:15, [PPEG]:[TDI] = 1:15 with 0.8%_wt_ ASC used as supports for 1-butyl-3-methylimidazolium hexafluorophosphate ([bmim][PF_6_]) and 1-ethyl-3-methylimidazolium bis(trifluoromethylsulfonyl)imide ([emim][Tf_2_N]) immobilization. The measured contact angles between the ILs on various polymeric supports are shown in Table 1. The capillary pressure was calculated using Young-Laplace equation (Equation 1), considering the average pore diameter measured by AFM:

*p*_c_ = (2γ cosθ)/*r* (1)

where *p*_c_ is the capillary pressure, Pa; γ, the surface tension (45.3 × 10^−3^ for [bmim][PF_6_]; 73.6 × 10^−3^ for [emim][Tf_2_N]), N/m; θ, contact angle, grad.; *r*, and the average pore radius, m.

**Table 1:** Contact angle of ionic liquids on polymeric supports, average pore diameter and calculated capillary pressure.

Polymeric Support	IL	Contact angle, º	Average Pore Diameter, nm	Capillary Pressure, bar
[PPEG]:[TDI] = 1:15 (co-catalyst CuCl_2_)	[bmim][PF_6_]	35 ± 1	11 ± 0.2	134.94 ± 0.08
[PPEG]:[TDI] = 1:15 (co-catalyst CuCl_2_) with 0.8%_wt_ ASC		57 ± 1	7.0 ± 0.2	141.98 ± 0.08
[PPEG]:[TDI] = 1:15 (co-catalyst CuCl_2_)	[emim][Tf_2_N]	49 ± 1	11 ± 0.2	175.58 ± 0.08
[PPEG]:[TDI] = 1:15 (co-catalyst CuCl_2_) with 0.8%_wt_ ASC		63 ± 1	7.0 ± 0.2	190.94 ± 0.08

The aforementioned equation points to the critical displacement pressure required to push the impregnating liquid out of the pores. The contact angle is one of the summands of the equation. According to this equation, the capillary pressure depends on the surface tension of liquid, the contact angle between the support and liquid, and the pore size of the support. The contact angle describes the interactions between the support and liquid phase, but the stability is also determined by other factors such as pore size. The smaller pore diameter for ASC modified polymer leads to a higher capillary pressure values. Thus, a higher stability is expectable for the sample with the highest capillary pressure, *i.e.*, ASC modified polymer.

Displacement occurs when the hydrostatic force exceeds the surface tension force holding the ionic liquid in support [43]. For the pressure values tested in this work (2 bar), the membranes are stable. Hence, the pressure difference is not the main cause of SILM degradation [44].

**Figure 7:**
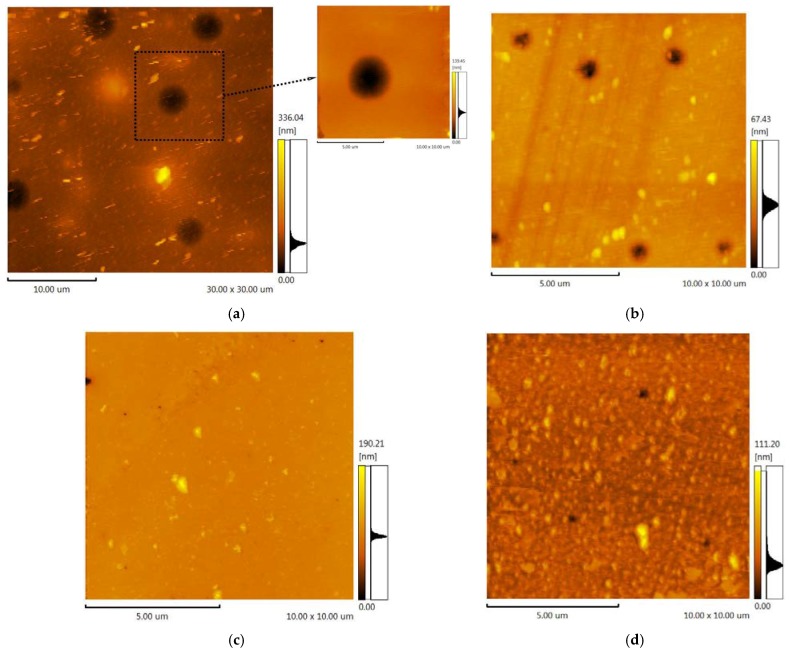
(**a**) AFM image (30 × 30 μm) of the unmodified polymer after the permeability test of SILM containing [emim][Tf_2_N]; (**b**) AFM image (10 × 10 μm) of the polymer modified by 0.8%_wt_ ASC after the permeability test of SILM containing [emim][Tf_2_N]); (**c**) AFM image (10 × 10 μm) of the unmodified polymer after the permeability test of SILM containing [bmim][PF_6_]; (**d**) AFM image (10 × 10 μm) of the polymer modified by 0.8%_wt_ ASC after the permeability test of SILM containing [bmim][PF_6_]).

The filling of ionic liquids into pores was investigated using AFM method. In Figure 7, the polymers surfaces after the IL immobilization and after gas permeation tests are presented. The figure illustrates that the sizable growth of average pore size occurs after the immobilization of [emim][Tf_2_N] and after the permeation test. The average pore diameter on the surface evaluated from 1450 to 3700 nm for the unmodified polymer, and from 400 to 1000 nm for the modified polymer. The low value of pores depth points to the presence of IL into polymeric supports. The change of pores diameter can be explained by the swelling of the polymers with [emim][Tf_2_N]. The roughness for SILMs based on the unmodified polymer (R_a_ = 6.67 ± 0.01 nm) and ASC modified one decreased (R_a_ = 2.90 ± 0.01 nm) in both cases.

The SILMs based on [bmim][PF_6_] show totally different behavior. All pores are filled completely as the absence of voids on SILMs surface indicates. No swelling and change of geometrical sizes of the polymers were observed. The roughness for SILMs containing [bmim][PF_6_] varied from R_a_ = 3.24 ± 0.01 nm for the unmodified polymer to R_a_ = 5.06 ± 0.01 nm for ASC modified support.

### 2.4. Gas Separation Properties

Permeabilities of He, N_2_, H_2_S, CO_2_, and NH_3_ through polymers and SILMs are presented in Table 2. The non-polar helium and hydrogen sulfide molecules have exhibited very low permeability—less than 0.13 Barrer, which is a limitation of the experimental set-up used. This observation could be explained by the incompatibility of non-polar gases and flexible polyethylene oxide-polypropylene oxide block. The low permeability of hydrogen sulfide is due to the lower value of the Gibbs energy of solvation in comparison with carbon dioxide [45]. According to the solution-diffusion model, the gas permeability through non-porous polymeric membranes is a product of the kinetic factor diffusion coefficient and the thermodynamic factor solubility. The absence of strong intermolecular interactions between the ionic liquid and gas molecules decreases the possibility of desorption of penetrant at the permeate side of the membrane. 

Various transport mechanisms can be considered for the porous polymeric supports. The viscous flow occurs in large pores (>10 μm), the gas molecules are transported jointly, and no separation is observed. In smaller pores, the size of which is comparable with the path of gas molecules, the Knudsen diffusion appears. The diffusion coefficient in the Knudsen regime depends on the molecule’s radius and the square root of molecular weight of gases [46]. Considering the mass-transfer through the porous polymeric membranes with very low amount and size of pores, the solution-diffusion mechanism and Fick’s first law as in the case of dense membranes must be considered. The gas permeation through a dense polymer membrane is proportional to solubility and diffusion coefficients, whereas the gas dissolution is based on Henry’s law, where the concentration of the gas in the membrane was directly proportional to the applied gas pressure [47]. The penetrations of gases through the SILMs and polymer matrixes have some specific features. The permeability of SILMs also can be explained by solution-diffusion mechanism of penetrants, only through the liquid phase [48]. The driving force is the concentration gradient between the different sides of membrane. The permeability of gases depends on the partition coefficient between the ionic liquid and feed/receiving phase.

The acidic gas CO_2_ is able to pass through all samples. The highest permeability was obtained with the polymeric support based on [PPEG]:[TDI] = 1:15 with 0.8%_wt_ ASC. The modified polymer presents a permeability almost two times larger than the unmodified. The immobilization of IL diminishes the permeability of CO_2_ in both cases.

The opposite tendency was observed for NH_3_ sorption. The unmodified polymer allowed for nearly twice the ammonia permeability. The presence of IL into polymer increases the NH_3_ permeability on the polymeric supports, and the permeation of NH_3_ through SILMs was increased by 30% and 76% for unmodified and modified samples, respectively. According to the results available in literature [49], ammonia is highly soluble in imidazolium-based IL due to the formation of strong hydrogen bonds with the ring hydrogen atoms of the cation.

It is well known that the diffusion coefficient exponentially decreases with the increase of the effective gas diameter. The effective diameters of gases diminish in the series He < H_2_S < CO_2_ < NH_3_ < N_2_. If the molecules’ dimensions have a significant contribution to the permeability results, a correlation should be observed between the size of gas molecules and permeability; however, no such relationship is seen. Thus, the molecules of helium and nitrogen correspond to the minimum and maximum values of the effective diameter and present a low permeability value. Ammonia molecules are larger than the molecules of CO_2_, and the permeability of ammonia is significantly higher as compared to carbon dioxide. Given these observations, the solubility of gases in the polymer must be considered and reveals low solubility for the polymers. The solubility is proportional to the applied pressure (Henry’s law) for elastomers, and is described by free volume model and Flory-Huggins thermodynamics. The high values of solubility were observed for the specific interactions in polymer-gas system. From this observation, it follows that the change of permeability of membrane at constant pressure is also determined by the specific interactions between the sorbent and gases. Therefore, when looking to gas permeability results, both gas diffusion and solubility are considered.

**Table 2:** Permeabilities of pure gases.

Polymeric Support	IL	Permeability, Barrer				
		He	N_2_	H_2_S	CO_2_	NH_3_
[PPEG]:[TDI] = 1:15 (co-catalyst CuCl_2_)	none	<0.13	3.0 ± 0.5	<0.13	200 ± 10	620 ± 10
[PPEG]:[TDI] = 1:15 (co-catalyst CuCl_2_) with 0.8%_wt_ ASC	none	<0.13	3.0 ± 0.5	<0.13	390 ± 24	370 ± 8
[PPEG]:[TDI] = 1:15 (co-catalyst CuCl_2_)	[bmim][PF_6_]	<0.13	<0.13	<0.13	100 ± 4	810 ± 12
[PPEG]:[TDI] = 1:15 (co-catalyst CuCl_2_) with 0.8%_wt_ ASC	[bmim][PF_6_]	<0.13	<0.13	<0.13	100 ± 4	650 ± 7
[PPEG]:[TDI] = 1:15 (co-catalyst CuCl_2_) with 0.8%_wt_ ASC	[emim][Tf_2_N]	-	4.0 ± 0.1	-	82.5 ± 0.4	-

It becomes apparent that the higher CO_2_ permeability for the unmodified membrane is related to the state of flexible-chain polyether phase. The dissolution and diffusion of gases occurs through the polyether component, and according to the measurement of dielectric losses, modification of the polymer refines the mobility of polyether chains and shifts the α-transition temperature to the low temperature region. This facet affects the rate of CO_2_ diffusion through the polyether phase.

The unmodified support has a rough surface as compared to the smooth ASC modified sample. This suggests the larger surface area of unmodified polymer comes in contact with IL, thereby increasing the effective surface area and stipulating higher gas transport properties and enhanced stability of SILM. On the other hand, the average pore size of unmodified polymers is larger than the ASC modified ones, meaning that the surface of pores on the unmodified membrane is less than the modified one. Both factors contribute to transport properties of supported ionic liquid membranes. The permeability data shows that the permeation of CO_2_ for unmodified support decreases two-fold, or for the modified support, by a factor of 3.9. Consequently, the pore size and distribution is more important for the transport properties of polymer than roughness.

In previous literature, CO_2_ permeation data for SILMs based on [bmim][PF_6_] [50,51,52,53] and [emim][Tf_2_N] [54,55,56,57] have been reported for using polyethersulfone (PES), alumina, and polyvinylidene fluoride (PVDF) supports (Table 3 and Table 4). The results obtained in our paper are comparable with the permeability of [bmim][PF_6_] immobilized on PVDF support [52]. The permeability of [emim][Tf_2_N] supported in studied polymers was slightly lower than the data reported in literature. 

There are several requirements for polymeric supports: they must be chemically inert in regards to IL; to be porous to allow for IL immobilization; to have affinity for IL and minimal IL penetration through the membrane and the transfer to other side of membrane; to exhibit the high mechanical and thermal stability. The affinity of the support and IL is determined by the chemical nature of polymer (the hydrophobic or hydrophilic nature). The hydrophobic ILs are more compatible with hydrophobic polymeric supports and *vice versa*. The compatibility is the substantial factor for the effective incorporation of IL into the support pores due to the low viscosity of ILs.

**Table 3:** The CO_2_ permeability through supported ionic liquid membranes (SILMs) based on [bmim][PF_6_].

Support	Pore Diameter, nm	Membrane Thickness, μm	Porosity, %	Permeability, Barrer	Reference
Hydrophilic polyethersulfone (Beijing Membrane Corporation)	220	150	80	1290	[50]
Alumina (Whatman Corporation)	20	60	25–50	800	[51]
Hydrophobic polyvinylidene fluoride (Millipore Corporation)	220	125	70	168.7	[52]
Hydrophobic polyvinylidene fluoride (Wako Pure Chemical Industries, Ltd.)	200	95	-	9800	[53]

**Figure 8:**
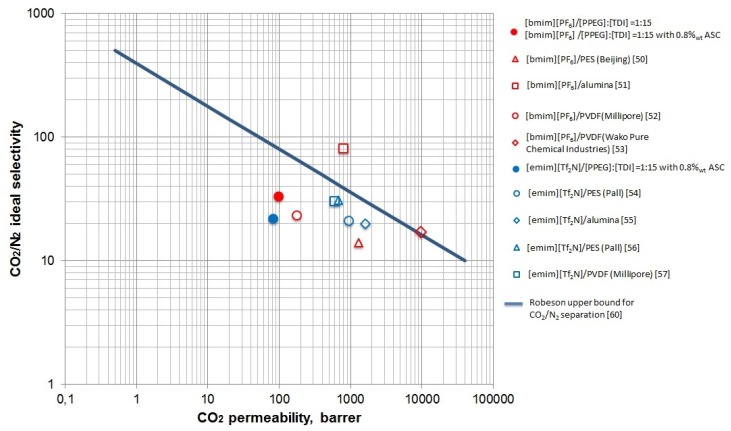
The Robeson plot for separation carbon dioxide from nitrogen.

Table 5 provides the calculated ideal selectivities for NH_3_ and CO_2_ in nitrogen, helium, or hydrogen sulfide, and NH_3_/CO_2_. [PPEG]:[TDI] = 1:15 [bmim][PF_6_] showed enhanced selectivity due to polymer matrix properties and IL presence for the ammonia separation. This result can be explained by polarity of ammonia and its thermodynamic compatibility with block copolymers of ethylene oxide and propylene oxide. Immobilization of IL improves the diffusion of polar gases and the solubility of ammonia most notably. The modified polymeric support was capable of separating polar gases only in the immobilized state; in the case of only the polymeric membrane, the permeabilities of CO_2_ and NH_3_ have the same values. Thus, we found lower permeability values are seen than are found in the literature [58,59]. According the literature data, the respective Robeson plot was formed (Figure 8). It demonstrates the current state of art in membrane technology for CO_2_/N_2_ separation, and the results obtained in this work are placed just below the upper bound. The selectivity of our SILMs are substantially consistent with the values for SILMs reported in the literature [50,51,52,53,54,55,56,57], the permeabilities of carbon dioxide investigated in this work were slightly smaller than reported by others.

**Table 4:** The CO_2_ permeability through SILMs based on [emim][Tf_2_N].

Support	Pore diameter, nm	Membrane thickness, μm	Porosity, %	Permeability, Barrer	Reference
Hydrophilic polyethersulfone (Pall Corporation)	200	152	-	960	[54]
Alumina (Whatman Corporation)	20	60	24	1620	[55]
Hydrophilic polyethersulfone (Pall Corporation)	200	145	80	680	[56]
Hydrophobic polyvinylidene fluoride (Millipore Corporation)	220	125	70	600	[57]

**Table 5:** Ideal selectivity for gas mixtures

Polymer support	Ideal Selectivity				
	NH_3_/G*	NH_3_/N_2_	CO_2_/G*	CO_2_/N_2_	NH_3_/CO_2_
[PPEG]:[TDI] =1:15 (co-catalyst CuCl_2_)	>4800	>4800	>1500	>1500	3.1 ± 0.2
[PPEG]:[TDI] =1:15 (co-catalyst CuCl_2_) with 0.8%_wt_ ASC	>2800	>2800	>3000	>3000	0.9 ± 0.1
[PPEG]:[TDI] =1:15 (co-catalyst CuCl_2_) [bmim][PF_6_]	>6200	270 ± 10	>770	33 ± 3	8.1 ± 0.3
[PPEG]:[TDI] =1:15 (co-catalyst CuCl_2_) with 0.8%_wt_ ASC [bmim][PF_6_]	>5000	217 ± 10	>770	33 ± 3	6.5 ± 0.3
[PPEG]:[TDI] =1:15 (co-catalyst CuCl_2_) with 0.8%_wt_ ASC [emim][Tf_2_N]	-	-	-	21.6 ± 2	-

* G—helium, hydrogen sulfide

## 3. Experimental Section 

Block copolymer of propylene oxide with ethylene oxide (PPEG) with molecular formula HO[CH_2_CH_2_O]_n_[CH_2_(CH_3_)CH_2_O]_m_[CH_2_CH_2_O]_n_K, where n ≈ 14 and m ≈ 48, molecular weight 4200 g/mol, content of potassium alcoholate groups is 10.9% from the total the number of functional groups, was purchased from PJSC Nizhnekamskneftekhim (Nizhnekamsk, Russia). Polyethylene glycol, molecular weight 400 g/mol (PEG), diethylene glycol (DEG-K), tetraethoxysilane (TEOS), toluene 2,4-diisocyanate (98% (TDI), CuCl_2_ (99.995% purity) and 1-butyl-3-methylimidazolium hexafluorophosphate ([bmim][PF_6_]) were purchased from Sigma-Aldrich (USA). 1-ethyl-3-methylimidazolium bis(trifluoromethylsulfonyl)imide ([emim][Tf_2_N]) was supplied by Io-Li-Tec, Ionic Liquids Technologies GmbH (Germany). The low molecular weight polydimethylsiloxane (PDMS) was purchased from Kazan synthetic rubber plant.

For the gas separation experiments, helium, nitrogen, and carbon dioxide were used with purity no less than 99.995% (NII KM, Russia). High purity ammonia 99.99999% was purchased and used as received (Firm HORST, Russia). Reaction media for the synthesis of block-copolymers was ethyl acetate. PPEG, PEG, and PDMS were dried at reduced pressure (~5 mmHg) and at elevated temperature of 353 K down to 0.01% moisture concentration. All other reagents and solvents were used without further purification.

The apparatus for ASC synthesis includes a round-bottom flask and an adapter with a stirrer. The synthesis was performed in three steps at elevated temperature of 90–92 °C. In the first step, a predetermined amount of PDMS was placed into the flask and heated to 90 °C. The PEG and TEOS were added to reaction mixture (20%_wt_ from the total amount of oligomers and TEOS), followed by stirring for one hour, at which point an additional portion of TEOS (10%_wt_ from the total amount of TEOS) and DEG-K (0.1%_wt_ from the mass of reaction mixture) was introduced in flask. In the final step, the residiual amount of TEOS was added to the reaction mixture. The obtained organic-inorganic modifier was placed in flat bottomed flask and dried at 120 °C for 1 hour with stirring before the viscosity of the reaction mixture becomes high.

Synthesis of polymers based on TDI and PPEG (as macroinitiator) was carried out in selected solvent (ethyl acetate) in presence of co-catalyst CuCl_2_. In the macroinitiator solution, the calculated amount of ASC as a modifier was injected at 298 K and under constant stirring, the reaction mixture was heated to 318 K. The calculated amount of toluene 2,4-diisocyanate was added. Upon completion of addition, the solution was stirred for five minutes at 318 K. The total content of the reactants in the solution was 25%. Eventually, the molar ratio of macroinitiators [TDI]:[PPEG] was 15. ASC content varied in the range of 0.1 to 15%_wt_.

The resulting solution of a polymer-forming system was cast in a laboratory dryer. The initial thickness of the cast film was adjusted to 600 µm using an applicator-casting knife. After the removal of the membrane from the dryer and allowed to dry cool at to room temperature, the material was characterized and used for gas permeation experiments. The thickness of the obtained polymeric films was in the range between 210–370 µm. 

For the preparation of the supported ionic liquid membranes, the pressure method was used. The polymeric support was fixed into a membrane module, and atop the polymer was placed a thin layer of ionic liquid. Then, a pressure of 2 bar was applied for filling the polymer pores by IL. After two hours, the pores of membrane were filled (according to gravimetric measurements) and the excess of IL was wiped by absorbing tissue. The uptake of IL by polymers is presented in Table 6.

**Table 6:** Mass uptake of the ionic liquids (IL) by the polymeric supports.

Polymer support	Ionic liquid	Uptake, % (w/w)
[PPEG]:[TDI] = 1:15 (co-catalyst CuCl_2_)	[bmim][PF_6_]	2.0
[PPEG]:[TDI] = 1:15 (co-catalyst CuCl_2_) with 0.8%_wt_ ASC	[bmim][PF_6_]	4.2
[PPEG]:[TDI] = 1:15 (co-catalyst CuCl_2_)	[emim][Tf_2_N]	51.9
[PPEG]:[TDI] = 1:15 (co-catalyst CuCl_2_) with 0.8%_wt_ ASC	[emim][Tf_2_N]	6.4

The summary pore volume of supports was investigated in accordance with Russian National Standard [61], and it was equal to 0.0525 and 0.051 cm^3^/g for the unmodified and modified samples, respectively. The pore filling values with the [bmim][PF_6_], defined as a relation of volume of IL to the support porous volume, were 27.6% and 59.6% for unmodified and modified polymers. With [emim][Tf_2_N], significant swelling was observed on the unmodified polymer. This was determined to be the cause for the unmodified polymer impropriety for gas separation measurements.

The stability of SILMs was determined by gravimetric method. The weights of SILMs before and after the N_2_ transport performances were compared. For the pressure values tested (2 bar), the membranes are stable, and no IL displacement from the polymeric support pores was detected.

The measurement of contact angle between the polymer and ionic liquid was provided using the drop shape method. The droplet of IL was placed on the polymer surface and an image of drop shape was obtained. The optical system apparatus consisted of a light source, an adjustable stage, and a USB optical microscope. The microscope (Chuo Seiki, TS-H) was fixed on an adjustable microscope mount. A digital image of the drop shape was made using CCD camera of microscope. The Image J software with Dropsnake plugin was used for calculation of contact angle value. 

The size of ASC particles was determined by Zetasizer Nano dynamic light scattering detector (Malvern Instruments Limited) equipped by He-Ne gas laser (wavelength of 633 nm) at ambient conditions. The signal analysis was carried out using single-board multi-channel correlator, paired with the IBM PC. The effective hydrodynamic radius of particles was calculated using the Stokes–Einstein equation. The solutions were filtered via Millipore filter for the removal of impurities. The measurement range was equal from 2 nm to several microns, providing an error of measurement of +/- 5%. 

The topography of membrane surface was determined by atomic force microscopy (AFM). Atomic force microscope Shimadzu SPM-9700 (Japan) with scanner 30 µm was used in the force modulation mode. As a tip, commercially available silicon tip POINTPROBE FMR-20 S/N-71814F8L882 (Nano World Innovative Technologies, Matterhorn, Switzerland) was used with spring stiffness 1.3 N/m and the radius of the curve of the tip typically not more than 8 nm and guaranteed to be not more than 12 nm. Scan sizes were 10 × 10 µm and 30 × 30 µm. The surface characterization was carried out at ambient temperature. The samples were cleaned of dust with ethanol before measurement, and then affixed to the center of the sample holder using a two-sided carbon tape (SPI Supplies Division of STRUCTURE PROBE Inc., West Chester, PA, USA). The determination of effective diameter of conical pore was performed by AFM measurements of the narrowest point of pores. This point was found on the topographic map of the surface with dimensions 2 × 2 mm^2^, which is represented on it as a “bottom” of the pores [41]. The cantilever with a curvature radius of tip not more than 8 nm was used for the observation of a topographic map to minimize the error introduced by the cantilever due to the narrowing of profile recesses. The results were statistically processed and the plots of pore size distribution were made. The type of obtained histograms was most similar to normal Gaussian distribution. The validity of the assumption, that the experimental samples have normal distribution, was mathematically supported using strict Pearson criterion, and the multiple criterion. After image acquisition the arithmetic average roughness height R_a_, was obtained by a program in the AFM image processing toolbox (SPM Online, Version 4.02, Shimadzu, Kyoto, Japan). The accuracies of obtained values were equal to 0.01 nm for average roughness R_a_. 

The SEM images were taken with Tescan Vega II electron microscope at the voltage of 10 kV with backscattered electron (BSE) detector. No special pretreatment for the samples was used. For the cross-section SEM-images, the membrane without ionic liquids was immersed in 0.1 M CsNO_3_ water solution, then the surface was cleaned by ethanol water solution before the samples were broken in liquid nitrogen.

**Figure 9:**
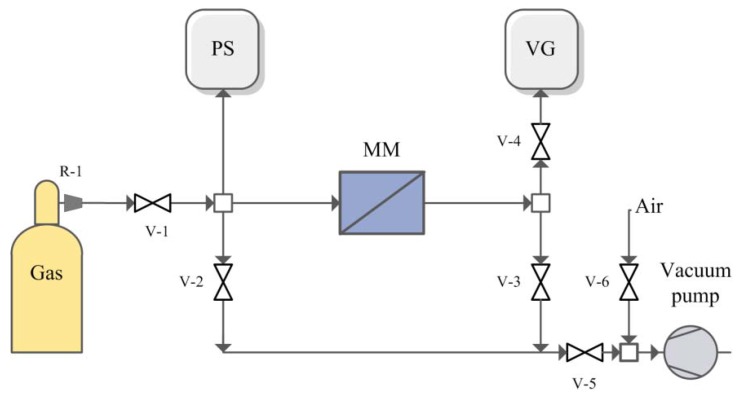
Scheme of experimental set-up for gases permeability measurements.

The single gas permeabilities of He, N_2_, CO_2_, H_2_S and NH_3_ through the obtained membranes were measured using a dead-end, vacuum regime [40,62] at ambient temperature with feed pressure of 2 bar. As a membrane module, a radial membrane module (MM, Figure 9) with a porous mechanical support [63] was used which was vacuum-degased before each measurement with the help of valves V-2, V-3 and V-5 which connected the apparatus to the vacuum pump. The effective surface of membrane was 14 cm^2^. The leak level was determined by a vacuum gauge (VG) after closing all valves before measurements. 

Gas reducer R-1 from cylinder, supplied the feed flow pressure. The upstream pressure level was recorded using a pressure sensor (PS). Pressure increase in the downstream side of a membrane module with time was recorded using a vacuum gauge (VG) when the valve V-3 was closed. The pressure of permeate was less than 10 mbar. The single gas permeabilities through every sample were measured not less than three times. After ammonia analysis, the permeability of helium and N_2_ was tested again to show the chemical stability of polymer. The errors of permeability measurements were less then 15%. After permeability measurements, the ideal selectivity was calculated as a ratio of permeability values.

Permeability coefficient *P* (1 Barrer = 3.348 × 10^−16^ mol·m·m^−2^·s^−1^·Pa) was calculated according to:

(2) $$P=\frac{V}{V_{m}}\frac{p_{2}T_{0}}{p_{0}T}\frac{l}{S\tau \left(p_{1}-p_{2}\right)}$$

where *V* is upstream volume; m^3^; *V*_m_, molar volume m^3^/mol; *p*_2_, downstream pressure for each individual experiment, Pa; *p*_1_, upstream pressure, Pa; *p*_0_, atmospheric pressure, Pa; *T*, temperature, K; *T*_0_, normal temperature, K; *S*, membrane area, m^2^; *l*, membrane thickness, m; τ, time of experiment, s. Thus, the permeability coefficient was submitted as mol·m·m^−2^·s^−1^·Pa. 

Ideal selectivity for pair of gases was calculated according to:

(3) $$\alpha =\frac{P_{A}}{P_{B}}$$

## 4. Conclusions

In this study, the method of synthesis of silica clusters associated in oligomeric media (ASC) on the basis of tetraethoxysilane, oligoethilene glycol, and oligodimethyl siloxane as thermodynamically incompatible oligomers was performed. According to the analysis of cumulative distribution function, the average ASC cluster size was equal to 20 nm. The microporous polymers were obtained via polyaddition of toluene 2,4-diisocyanate to anionic macroinitiators in the presence of organic-inorganic modifiers. The polymers exhibited high ability to immobilize the ionic liquids due to the specific structure of the inner surface of pores.

The results of atomic force microscopy found out that the organic-inorganic modifiers had a significant impact on the microphase separation in the polymer matrix. The ionic liquids [bmim][PF_6_] and [emim][Tf_2_N] were immobilized into pores of modified and unmodified polymer. The smaller pore diameter for ASC modified polymer leads to higher capillary pressure values. Thus, a higher stability is expected for the sample with the highest capillary pressure, *i.e.*, ASC modified polymer.

The tested SILM membrane results showed promising results for hazardous gases separation processes. The modification of SILMs by nanosize silica particles leads to an increase of NH_3_ separation relative to CO_2_. 
